# Functional Differences in the Affected Limbs Between CPAK Type I and Type II Patients Undergoing Unilateral Total Knee Arthroplasty

**DOI:** 10.3390/medicina62020259

**Published:** 2026-01-26

**Authors:** Ju Won Bae, Seung Ik Cho, Dhong Won Lee, Byung Sun Park, Yu Bin Lee, Wonjun Chang, Joon Kyu Lee

**Affiliations:** Department of Orthopaedic Surgery, Konkuk University Medical Center, Seoul 05030, Republic of Korea; bird200503@naver.com (J.W.B.); bestjjo@hanmail.net (S.I.C.); wonbayo@naver.com (D.W.L.); qud516@naver.com (B.S.P.); 20240031@kuh.ac.kr (Y.B.L.); jwj9967@naver.com (W.C.)

**Keywords:** total knee arthroplasty, CPAK, time up and go test, 4 m walk test, isokinetic knee muscle strength, hip abductor strength, dynamic balance

## Abstract

*Background and Objectives:* This study compared functional performance, gait performance, and dynamic balance between Coronal Plane Alignment of the Knee (CPAK) Type I and Type II patients undergoing unilateral total knee arthroplasty (TKA). *Materials and Methods:* We included 162 consecutive patients scheduled for unilateral TKA between January 2022 and August 2025. Patients were classified according to CPAK type; 42 were Type I and 33 were Type II. Preoperative assessments included demographic data, Korean Knee Score, and WOMAC. Functional performance was evaluated using the Timed Up and Go (TUG) test and the 4 m walk test. Isokinetic knee extensor and flexor strength (60°/s), hip abductor strength, and bilateral thigh circumferences measured 5 cm and 15 cm proximal to the patella were assessed. Dynamic balance asymmetry was evaluated using the POSTUROMED device. Inter-limb differences were calculated by comparing the operated and non-operated limbs. *Results:* No significant between-group differences were observed in clinical scores, knee extensor, or flexor strength deficits, hip abductor strength deficits, or thigh circumference differences. However, CPAK Type II patients demonstrated significantly better functional performance, with faster TUG (*p* = 0.014) and 4 m walk test times (*p* = 0.022). Dynamic balance outcomes were also significantly better in the Type II group (*p* = 0.042). *Conclusions:* Despite similar patient-reported clinical scores and muscle strength, patients with the CPAK Type II phenotype exhibited superior gait performance and dynamic balance compared with those with Type I following unilateral TKA.

## 1. Introduction

Knee osteoarthritis (KOA) is one of the most common degenerative joint diseases worldwide. It has a high prevalence among the elderly and is a major cause of reduced quality of life. In Korea, the prevalence reaches approximately 36.1%, with women exhibiting about 1.68 times higher rates than men [1]. KOA arises from various risk factors such as obesity, muscle weakness, trauma, and lower limb malalignment. It is a chronic condition characterized by cartilage degeneration, secondary osteophyte formation, synovitis, and changes in the ligaments and joint capsule [2]. Recent large-scale clinical reviews have further emphasized the substantial global burden of KOA and its significant impact on functional performance and quality of life, highlighting the importance of incorporating updated evidence into contemporary OA research [3].

Patients with KOA experience not only pain but also functional performance limitations such as reduced lower limb strength, impaired proprioception, and unstable gait [4]. Quadriceps and hip abductor muscle weakness is common; quadriceps strength has been reported to decline by 20~40%, depending on severity [5]. This muscle weakness can lead to decreased ability in daily activities, increased risk of falls, and altered gait patterns [6]. Recent studies have also reported that objective functional assessments, including gait performance and balance analysis, provide valuable information for evaluating disease severity and functional impairment in patients with KOA [7]. The Kellgren–Lawrence (KL) grading system, widely used to assess KOA progression and prognosis, reflects increasing lower limb malalignment that may lead to further functional decline [8]. For this reason, lower limb alignment assessment is often performed in patients with KOA [9,10]. Malalignment, such as varus or valgus alignment, can cause uneven load distribution in the joint, thereby accelerating disease progression [11]. In particular, varus alignment concentrates the load on the medial compartment of the joint, promoting cartilage wear [12].

Historically, knee alignment was classified as varus, valgus, or neutral based on the mechanical axis on long-leg radiographs. However, this approach is insufficient to reflect the complexity of coronal alignment patterns [8,12].

Recently, to allow more precise analysis of coronal alignment, MacDessi and colleagues proposed the Coronal Plane Alignment of the Knee (CPAK) classification system. This system is based on the arithmetic hip–knee–ankle angle (aHKA) and joint line obliquity (JLO) and categorizes knees into nine types. Unlike the traditional varus/valgus/neutral classification, CPAK offers a more individualized approach to surgical planning [12,13]. Although the CPAK classification has been increasingly adopted to describe constitutional knee alignment, its clinical utility and validity in predicting functional outcomes remain controversial. Recent studies have reported limited reproducibility and questioned whether CPAK phenotypes can reliably reflect clinically meaningful functional differences in patients with knee osteoarthritis and after total knee arthroplasty [14,15,16].

To date, there are limited quantitative analyses of functional performance such as limb strength and balance based on CPAK classification. Few studies have investigated the functional differences according to CPAK types, especially in patients with advanced KOA. Recent findings suggest that CPAK Type I is the most prevalent phenotype as the disease progresses [17,18], highlighting the need to explore functional performance differences among CPAK types. This could provide meaningful insights for optimizing treatment strategies.

This study aimed to compare functional performance, gait performance, and dynamic balance differences between CPAK Type I and Type II patients undergoing unilateral total knee arthroplasty (TKA). The goal was to better understand the functional performance limitations associated with each CPAK type and offer ground data for effective rehabilitation and treatment strategies. We hypothesized that functional performance and dynamic balance would differ between CPAK Type I and Type II phenotypes in patients undergoing unilateral total knee arthroplasty.

## 2. Materials and Methods

### 2.1. Study Design and Participants

**Figure 1:**
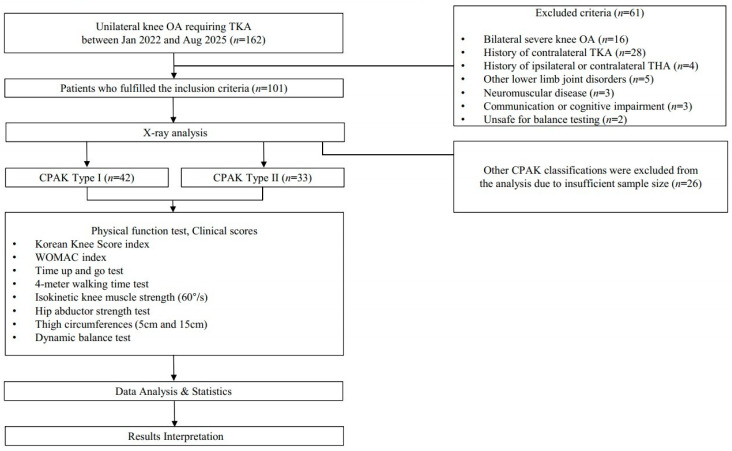
Flowchart of the patient selection process. OA, osteoarthritis; TKA, total knee arthroplasty; THA, total hip arthroplasty; WOMAC, Western Ontario and McMaster Universities Arthritis Index; CPAK, Coronal Plane Alignment of the Knee.

This study was a retrospectively designed comparative analysis. One hundred and sixty-two consecutive patients who were scheduled to undergo unilateral TKA (defined as having pain in only one knee severe enough to require TKA) at our institution between January 2022 and August 2025 were included in this study. The exclusion criteria were as follows: (1) severe bilateral OA on radiographs, (2) history of contralateral TKA, (3) history of ipsilateral or contralateral total hip arthroplasty, (4) other lower limb disorders such as neuromuscular disease, (5) communication or cognitive impairment, (6) unsafe for the dynamic balance test. Of the 162 patients, 61 met at least one of the exclusion criteria. Among the remaining 101 patients, 42 were classified as CPAK Type I and 33 as Type II. The other 26 patients, who belonged to different CPAK types, were excluded because their sample sizes were insufficient. Patients classified as CPAK Type I or II were included. Figure 1 presents a detailed flowchart of the study design, including patient inclusion, grouping, and testing procedures.

This retrospective study was conducted in accordance with the Declaration of Helsinki and approved by the institutional review board of Konkuk University Medical Center (KUMC 2025-06-055, on 30 June 2025). Informed consent was obtained from all enrolled patients either directly or over the phone.

### 2.2. Demographic Data and Clinical Scores

Demographic data were collected and used for comparative analysis between the two groups. Collected data included age, sex, height (cm), weight (kg), body mass index (BMI), and operated side (left or right). There were no statistically significant differences in baseline demographic characteristics between the groups (Table 1). Two knee scores (the Korean Knee Score (KKS) and the Western Ontario and McMaster Universities Arthritis Index (WOMAC)) were obtained.

### 2.3. Radiographic Evaluation

All participants underwent full weight-bearing anteroposterior long-leg standing radiographs of the lower limbs to assess coronal plane alignment. Key radiographic measures included the medial proximal tibial angle (MPTA), lateral distal femoral angle (LDFA), aHKA (MPTA − LDFA), and JLO (MPTA + LDFA). CPAK classification was applied based on the criteria established by MacDessi et al. in 2021 [12].

### 2.4. Strength and Functional Performance Assessments

All measurements were performed by experienced examiners who were trained in standardized testing protocols prior to data collection. The measuring devices were calibrated according to the manufacturers’ guidelines before each testing session. Identical procedures and instructions were applied to all participants to ensure consistency across assessments. When applicable, the same examiner conducted repeated measurements for each participant to minimize examiner-related differences. These procedures were implemented to enhance the reliability and reproducibility of the measurements.

#### 2.4.1. Lower Limb Strength Measurement

**Figure 2:**
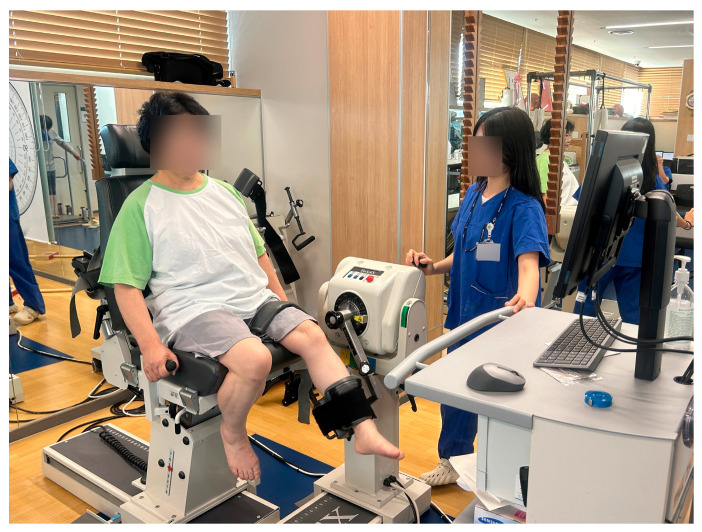
Assessment of isokinetic knee extension and flexion strength using the Biodex System 4 Pro dynamometer.

Isokinetic knee strength was measured with the System 4 Pro dynamometer from Biodex Medical Systems (Shirley, NY, USA). Participants were seated and positioned so that the center of the lateral femoral epicondyle aligned with the dynamometer’s axis of rotation. The range of motion was restricted to 0°~90° to prevent excessive flexion or extension. Knee extension and flexion were assessed at an angular velocity of 60°/s, with two trials per movement (Figure 2). The peak torque value from the trials was recorded. Strength differences between the operated and non-operated limbs were expressed as percentages.

**Figure 3:**
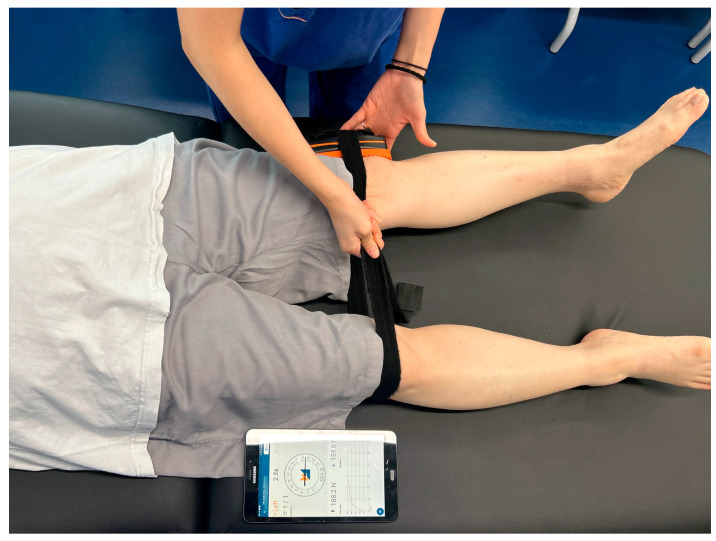
Assessment of isometric hip abductor strength using the K-Myo handheld dynamometer.

Hip abductor strength was measured with the K-Myo handheld dynamometer (Kinvent Biomecanique, Montpellier, France). Participants lay supine with both legs shoulder-width apart. A strap was secured around the lateral femoral epicondyle, and participants performed a 5 s maximal isometric hip abduction [19] (Figure 3). The output was recorded in Newtons, and inter-limb differences were calculated as a ratio.

#### 2.4.2. Gait Performance Measurement

**Figure 4:**
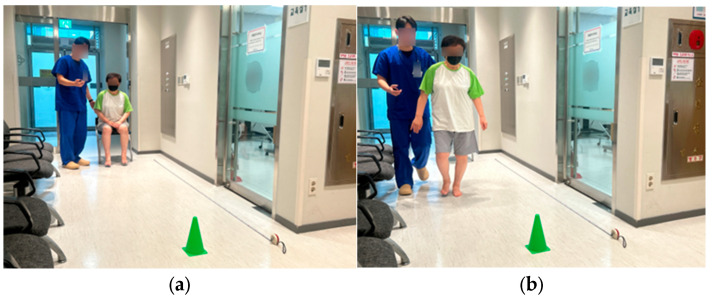
Timed Up and Go (TUG) test: (**a**) starting position with the participant seated in a standard chair before initiating the TUG test; (**b**) mid-test phase showing the participant walking during the TUG test.

Gait performance function was assessed using the Timed Up and Go (TUG) test and the 4 m walk test. For the TUG test, participants rose from a chair, walked 3 m, turned, returned, and sat down (Figure 4).

**Figure 5:**
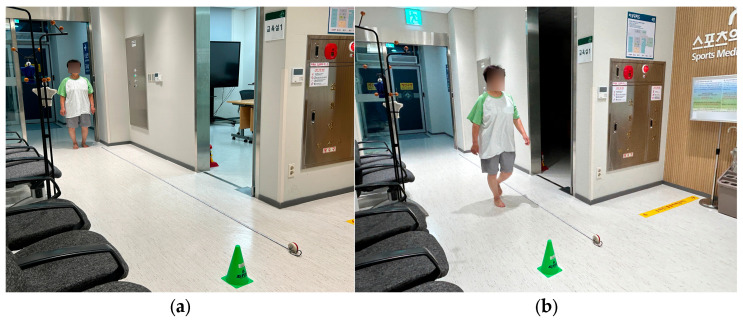
The 4 m walk test: (**a**) starting position with the participant standing at the designated starting line prior to initiating the 4 m walk test; (**b**) mid-test phase showing the participant walking along the straight 4 m path.

The time taken was measured to 0.01 s precision [20]. For the 4 m walk test, participants walked a straight 4 m course on command, and the time to complete the walk was recorded [21] (Figure 5).

#### 2.4.3. Thigh Circumference Measurement

**Figure 6:**
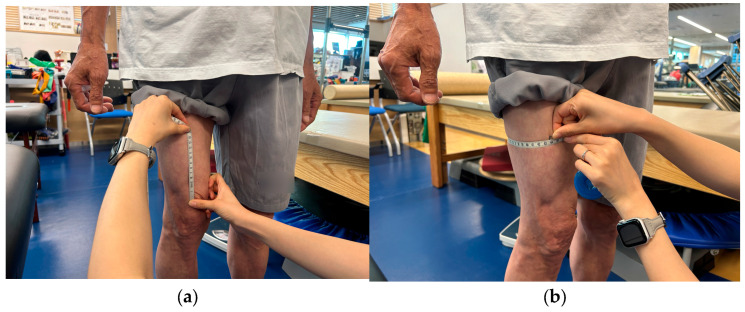
Thigh circumference measurement: (**a**) measurement of the proximal 15 cm distance from the superior border of the patella; (**b**) measurement of thigh circumference 15 cm proximal to the superior border of the patella.

Thigh circumference was measured based on the method described by Mathur et al. [22]. Participants stood upright with feet shoulder-width apart and weight evenly distributed. The circumference was measured with a tape at 5 cm and 15 cm proximal to the superior border of the patella (Figure 6). Measurements were taken on both limbs, and inter-limb differences were compared.

#### 2.4.4. Dynamic Balance Measurement

**Figure 7:**
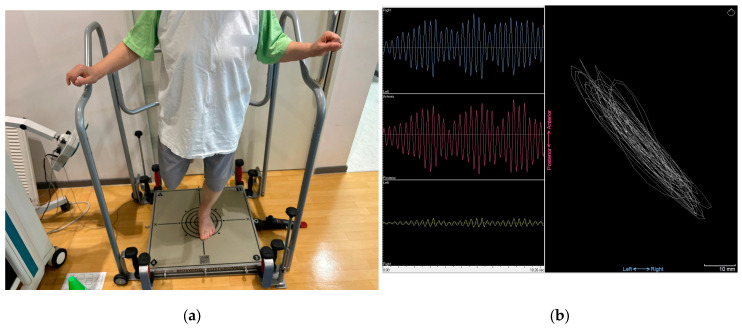
Dynamic balance measurement: (**a**) single-leg stance balance assessment on the POSTUROMED platform; (**b**) sway path recorded by the MicroSwing 6 accelerometer: blue line indicates mediolateral (left-right) sway, red line indicates anteroposterior sway, yellow line indicates rotational sway, and white line indicates total sway.

Dynamic balance was evaluated using the POSTUROMED platform (Haider Bioswing, Pullenreuth, Germany). Participants stood barefoot at the center of the platform with their second toes aligned to the reference line. With the knee flexed to approximately 30° and eyes open, participants performed a single-leg stance on the affected limb. Once stabilized, they released their hands and attempted to minimize sway for 10 s [23]. The sway path (in millimeters) was recorded by the MicroSwing 6 accelerometer installed beneath the device (Figure 7). Differences between the affected and unaffected limbs were expressed as percentages. The instability surplus percentage was used as an indicator of dynamic balance asymmetry, with higher values representing greater postural sway and poorer dynamic balance, and lower values indicating better postural control.

### 2.5. Statistical Analysis

All statistical analyses were performed using SPSS software version 24.0 (IBM Corp., Armonk, NY, USA). The Shapiro–Wilk test was used to check the data for normality. Comparisons between the groups were performed using the Student *t* test for continuous normal distribution data and the Mann–Whitney U test for ordinal categorical and non-normal distribution data. A Pearson chi-square test was used for nominal categorical data. The level of significance was set at *p* ≤ 0.05. A post hoc power analysis was performed using G*Power version 3.1.2 to assess the validity of the sample size based on the comparison of the TUG test. A post hoc power analysis showed that the sample size of 75 patients (42 Type I and 33 Type II) revealed adequate statistical power (0.86) based on the comparison of the TUG test.

## 3. Results

### 3.1. Clinical Scores

The KKS and the WOMAC showed no significant differences between the groups (*p* = 0.638 for the KKS, *p* = 0.441 for the WOMAC) (Table 2).

### 3.2. Muscle Strength and Thigh Circumference

There were no significant between-group differences in knee extensor or flexor strength deficits nor in hip abductor strength deficits (all *p* > 0.05) (Table 3). Differences in thigh circumference measured 5 cm and 15 cm proximal to the patella also did not differ significantly between groups (all *p* > 0.05) (Table 3).

### 3.3. Gait Performance and Dynamic Balance Outcomes

In contrast, patients with CPAK Type II showed significantly better functional performance and dynamic balance. In the TUG test, they completed the task significantly faster than the Type I group (*p* = 0.014). The 4 m walk test yielded similar results, with the Type II group recording faster times than the Type I group (*p* = 0.022). In the dynamic balance test using the POSTUROMED system, the Type II group showed a significantly lower instability surplus score than the Type I group, indicating reduced postural sway and better dynamic balance (*p* = 0.042) (Table 3).

## 4. Discussion

The most important finding of the present study was that patients with the CPAK Type II phenotype demonstrated superior gait performance and dynamic balance compared with those with the Type I phenotype, despite similar patient-reported clinical scores and muscle strength.

There were no significant differences in KKS or WOMAC between CPAK Types I and II. These clinical scores may reflect broader influences beyond alignment, such as compensatory strategies and preserved contralateral limb function. Isokinetic testing showed no significant between-group differences in muscle strength deficits of the affected limb for the knee flexors, knee extensors, or hip abductors. Previous studies support that malalignment alone may not impair muscle function, likely due to neuromuscular compensation [23]. Contrary to expectations, thigh circumference did not differ between types. This may be due to fat infiltration masking actual muscle atrophy [24], and, as Pedroso et al. noted, muscle strength does not always correlate with muscle mass [25]. Hence, thigh circumference may not reflect subtle alignment-related changes.

However, CPAK Type I patients showed significantly poorer dynamic balance based on higher POSTUROMED instability scores. The higher instability surplus percentage in the CPAK Type I group indicates greater postural sway and poorer postural control during the single-leg stance, reflecting reduced dynamic balance stability. This may stem from varus alignment shifting the mechanical axis medially, increasing medial joint loading and destabilizing weight distribution during movement. Greater lateral laxity and medial stiffness [10], combined with reduced proprioceptive input and delayed reflexes [26,27], can impair postural control and increase fall risk. Gait performance was also reduced in CPAK Type I, with slower TUG and 4 m walking speeds. A reduced MPTA, a key feature of Type I, shifts loading medially and prompts compensatory gait strategies such as shorter steps or slower speed. While adaptive, these strategies increase the knee adduction moment (KAM), worsening joint stress [11]. These findings suggest that gait dysfunction in CPAK Type I reflects both structural and neuromuscular adaptations that reduce efficiency and dynamic stability.

Although clinical scores and muscle strength were comparable between groups, patients with CPAK Type I demonstrated clear functional performance disadvantages in both dynamic balance and gait performance. These findings suggest that rehabilitation should extend beyond traditional strengthening to include targeted proprioceptive training and gait-specific training aimed at correcting biomechanical inefficiencies and neuromuscular control deficits, thereby supporting more complete recovery and reducing fall risk. In addition to mechanical explanations for the functional performance decline observed in CPAK Type I, alignment-tailored rehabilitation strategies merit consideration [12]. Varus alignment is consistently associated with increased medial compartment loading and an elevated KAM, both of which predict symptom exacerbation and structural progression [28]. For this subtype, interventions that reduce KAM or enhance frontal-plane stability are likely to be beneficial.

Combined strengthening of the hip abductors with conventional quadriceps training has been reported to improve pain and function in patients with varus-related medial OA, likely by decreasing medialization of the weight-bearing line, reducing medial compartment loading and KAM, and improving pelvic stability [29,30]. Furthermore, the addition of task-oriented neuromuscular and dynamic balance training, such as step-down exercises on unstable or frontal-plane challenging surfaces and perturbation-based drills, may help compensate for the proprioceptive deficits and dynamic instability that are more pronounced in CPAK type I phenotype. Several studies have demonstrated that balance and neuromuscular training can improve functional performance outcomes as effectively as strength training [31,32]. Considering that gait and functional performance assessment results in this study (e.g., TUG, 4 m walk, and dynamic balance tests) appeared to be more sensitive to alignment differences than to strength deficits, it would be appropriate for preoperative rehabilitation programs in TKA candidates with CPAK type I phenotype to incorporate alignment-specific exercises rather than relying solely on general strengthening routines.

Nevertheless, these findings should be interpreted with caution, as the clinical utility and reproducibility of the CPAK classification system remain controversial. Previous studies have questioned whether CPAK phenotypes consistently reflect clinically meaningful functional differences, which may influence the interpretation of alignment-based functional comparisons [14,15,16].

This study has several limitations. First, due to its retrospective design, causal relationships between CPAK phenotype and functional performance outcomes cannot be established. In addition, several potentially important confounding factors, including pain intensity, radiographic disease severity, physical activity level, and comorbidities, were not controlled for, which may have influenced the results. Second, this study was conducted at a single center with a relatively limited sample size, and only CPAK Type I and II phenotypes were analyzed because other CPAK types had insufficient sample sizes for reliable statistical comparison. Therefore, the generalizability of the findings to other CPAK phenotypes or broader populations may be limited. In addition, the inherent limitations of the CPAK classification system should be acknowledged. Although CPAK provides a structured framework for describing coronal knee alignment, its reproducibility and clinical predictive validity remain controversial, which may limit the interpretation and generalizability of the present findings. Third, this study focused exclusively on preoperative functional performance characteristics and did not evaluate postoperative changes in CPAK type or alignment following total knee arthroplasty. Postoperative alignment can vary depending on surgical technique, implant positioning, and soft-tissue balancing, and its influence on gait performance, dynamic balance, and rehabilitation outcomes remains unclear. Eventually, different CPAK phenotypes may require distinct postoperative rehabilitation strategies; however, this study did not investigate phenotype-specific rehabilitation approaches. Future prospective, multicenter studies with postoperative follow-up are needed to clarify alignment-related functional performance recovery patterns and to establish alignment-based rehabilitation protocols.

## 5. Conclusions

In patients undergoing unilateral total knee arthroplasty, those with the CPAK Type II phenotype demonstrated better gait performance and dynamic balance compared with those with the CPAK Type I phenotype, while patient-reported clinical scores and muscle strength were comparable between groups. These findings suggest that CPAK phenotype may be associated with functional performance and postural control rather than with subjective clinical symptoms or isolated muscle strength deficits. Therefore, assessment of CPAK phenotype may provide additional information for preoperative functional performance evaluation and for planning individualized rehabilitation strategies.

## Figures and Tables

**Table 1:** Comparison of demographics between the groups.

		Type 1 (*n* = 42)	Type 2 (*n* = 33)	*p* Value
Sex (Male/Female)		8/34	8/25	0.586 ^a^
Knee (Right/Left)		18/24	19/14	0.206 ^a^
KL grade (1/2/3/4)	Involved	0/0/9/33	0/0/12/21	0.153 ^a^
	Uninvolved	5/30/6/1	6/23/4/0	0.716 ^a^
Age * (Years)		70.0 ± 7.6	69.3 ± 4.7	0.700 ^b^
Body Mass Index * (kg/m^2^)		28.1 ± 4.8	26.2 ± 4.0	0.160 ^b^

^a^ Chi-square test, ^b^ Student’s *t*-test. * The values are given as the mean and the standard deviation. KL, Kellgren–Lawrence.

**Table 2:** Comparison of the clinical scores between the groups.

	Type 1 (*n* = 42)	Type 2 (*n* = 33)	*p* Value
Korean Knee Score	38.1 ± 12.5	36.2 ± 14.9	0.638 ^a^
WOMAC	48.8 ± 13.9	45.5 ± 16.4	0.441 ^a^

^a^ Student *t* test. The values are given as the mean and the standard deviation. WOMAC, Western Ontario and McMaster Universities Arthritis Index.

**Table 3:** Comparison of the functional performance, dynamic balance, and muscle strength between the groups.

	Type 1 (*n* = 42)	Type 2 (*n* = 33)	*p* Value
TUG test (seconds)	11.8 ± 6.7	8.6 ± 1.8	0.014 ^b^
4 m walking time test (seconds)	5.1 ± 2.8	3.8 ± 1.3	0.022 ^b^
Dynamic balance (instability surplus %)	205.4 ± 101.3	156.0 ± 68.3	0.042 ^b^
Knee extensor muscle strength difference (%)	37.8 ± 20.8	32.5 ± 19.8	0.366 ^a^
Knee flexor muscle strength difference (%)	25.5 ± 36.1	22.0 ± 26.0	0.702 ^a^
Hip abductor strength difference (%)	19.5 ± 14.8	14.7 ± 10.3	0.237 ^a^
Thigh circumference difference (5 cm from upper pole or patella, cm)	2.6 ± 4.0	0.2 ± 4.6	0.164 ^b^
Thigh circumference difference (15 cm from upper pole or patella, cm)	2.6 ± 4.7	2.4 ± 3.3	0.666 ^b^

^a^ Student *t* test, ^b^ Mann–Whitney test. The values are given as the mean and the standard deviation. TUG, Timed Up and Go test.

## Data Availability

The datasets analyzed in this study are not publicly available but are available from the corresponding author on appropriate request.
